# Racial Composition of Social Environments Over the Life Course Using the Pictorial Racial Composition Measure: Development and Validation Study

**DOI:** 10.2196/55461

**Published:** 2024-08-08

**Authors:** Jemar R Bather, Kimberly A Kaphingst, Melody S Goodman

**Affiliations:** 1 Center for Anti-racism, Social Justice & Public Health School of Global Public Health New York University New York, NY United States; 2 Department of Biostatistics School of Global Public Health New York University New York, NY United States; 3 Huntsman Cancer Institute University of Utah Salt Lake City, UT United States; 4 Department of Communication University of Utah Salt Lake City, UT United States

**Keywords:** racial residential segregation, racial composition, health equity, social environment, place, neighborhood composition, health inequities, social determinants of health

## Abstract

**Background:**

Studies investigating the impact of racial segregation on health have reported mixed findings and tended to focus on the racial composition of neighborhoods. These studies use varying racial composition measures, such as census data or investigator-adapted questions, which are currently limited to assessing one dimension of neighborhood racial composition.

**Objective:**

This study aims to develop and validate a novel racial segregation measure, the Pictorial Racial Composition Measure (PRCM).

**Methods:**

The PRCM is a 10-item questionnaire of pictures representing social environments across adolescence and adulthood: neighborhoods and blocks (adolescent and current), schools and classrooms (junior high and high school), workplace, and place of worship. Cognitive interviews (n=13) and surveys (N=549) were administered to medically underserved patients at a primary care clinic at the Barnes-Jewish Hospital. Development of the PRCM occurred across pilot and main phases. For each social environment and survey phase (pilot and main), we computed positive versus negative pairwise comparisons: *mostly Black versus all other categories, half Black versus all other categories,* and *mostly White versus all other categories*. We calculated the following validity metrics for each pairwise comparison: sensitivity, specificity, correct classification rate, positive predictive value, negative predictive value, positive likelihood ratio, negative likelihood ratio, false positive rate, and false negative rate.

**Results:**

For each social environment, the mostly Black and mostly White dichotomizations generated better validity metrics relative to the half Black dichotomization. Across all 10 social environments in the pilot and main phases, mostly Black and mostly White dichotomizations exhibited a moderate-to-high sensitivity, specificity, correct classification rate, positive predictive value, and negative predictive value. The positive likelihood ratio values were >1, and the negative likelihood ratio values were close to 0. The false positive and negative rates were low to moderate.

**Conclusions:**

These findings support that using either the *mostly Black versus other categories* or the *mostly White versus other categories* dichotomizations may provide accurate and reliable measures of racial composition across the 10 social environments. The PRCM can serve as a uniform measure across disciplines, capture multiple social environments over the life course, and be administered during one study visit. The PRCM also provides an added window into understanding how structural racism has impacted minoritized communities and may inform equitable intervention and prevention efforts to improve lives.

## Introduction

### Background

Research investigating the impact of racial segregation on health has typically focused on neighborhood racial composition [1-9]. Some scholars argue that this is due to racial segregation being an essential driver of health disparities [8,10]. However, evidence demonstrates the potential positive impacts of racial segregation [11,12]. For example, in a North Carolina study of Black church members, living in areas with a high proportion of Black individuals was associated with high physical activity [12]. Another example can be found in a study of New York City residents that demonstrated lower rates of all-cause mortality, coronary heart disease, and cardiovascular disease among older racialized Black people living in areas predominantly populated by Black people [13]. These mixed findings may result from different measures of racial segregation used across studies.

Most studies have used census data [1-5,7,8] or investigator-adapted questions [14-18] to measure racial segregation. Census data allow researchers to calculate the percentage of Black individuals in a census region and use this as an independent variable in regression analyses. Others have operationalized census data to construct local segregation-based measures such as the Index of Concentration at the Extremes [19,20], the dissimilarity index [21,22], the isolation index [23,24], the interaction index [23,25], the location quotient of residential segregation [26,27], and the Getis-Ord Gi* statistic [28].

Although census data can objectively measure neighborhood composition, their geographical ranges may be too broad and may not represent what study participants perceive as their neighborhood [16,29]. Therefore, some researchers use investigator-adapted survey items instead of census data to measure racial segregation [14-18]. Perceived neighborhood racial composition (PNRC) is an investigator-adapted and self-reported measure of the racial composition of a participant’s community [14]. The PNRC measure is limited in several ways. First, it focuses on a single neighborhood aspect [15]. Second, it does not address the difference that an individual may experience on a daily basis when moving between their home and school (or work) [16]. Moving between neighborhoods with varying levels of racial segregation can impact individual outcomes [30,31]. Proximity to predominantly White neighborhoods provides access to better public resources such as parks and pools and lower crime rates [30,31]. In contrast, predominantly Black neighborhoods often remain underdeveloped and underresourced [30,31]. However, proximity to predominantly White neighborhoods may also increase exposure to anti-Black discrimination such as racial profiling [30,31]. Finally, how it is asked on surveys varies across studies.

A measure that can account for differences across multiple social environments at different life stages will be helpful to social scientists. Analyzing the racial composition of social environments over the life course may help in identifying a susceptible period of racial segregation that has a more pronounced deleterious health effect than other periods [32-34]. This can guide public health efforts and interventions aimed at dismantling structural racism, broadening the understanding of psychosocial determinants of health, and elucidating the effects of racism at different developmental stages among populations considered marginalized [35-43].

Another limitation of census-based measures is the lack of validation in populations with low health literacy. Improving health literacy has become a global public health concern prioritized by clinicians, genetic counselors, and health communication specialists [44-46]. Limited health literacy has been associated with increased rates of emergency department visits, unintentional medication nonadherence, and all-cause mortality [47-55]. Creating a visual assessment of the racial composition of social environments provides researchers with an accessible survey measure to use in low health literacy populations, potentially improving patient care, engagement, and education.

An extension of the PNRC approach is needed that can (1) serve as a uniform measure across disciplines, (2) capture multiple social environments, (3) measure the racial composition of these social environments during 1 study visit, and (4) accommodate participants with limited health literacy. Measuring the racial composition over the life course is possible with census data and the PNRC measure. However, both approaches would require a longitudinal design, which is susceptible to participant dropout. Measuring the racial composition of life course environments is crucial because racial segregation is multidimensional, interconnected with racism, operates across various social settings, and has cumulative effects [10,30,56,57].

### Objective

We propose a novel approach to measuring the racial composition of social environments over the life course. The Pictorial Racial Composition Measure (PRCM) is a 10-item questionnaire of pictures representing social environments across adolescence and adulthood. The PRCM is intended to differentiate the racial composition of multiple social environments. We develop and validate the PRCM using data from cognitive interviews and surveys administered to a medically underserved patient population. The written version of this racial composition measure was previously validated against census data [14] and has been applied to study the impact of racial segregation on hypertension diagnosis [58] and health literacy [59,60]. This study aimed to validate the pictorial version against the written version.

## Methods

### Setting

This study was conducted at the Center for Outpatient Health (COH) primary care clinic at the Barnes-Jewish Hospital.

### Ethical Considerations

The Human Research Protection Office at the Washington University School of Medicine approved the primary data collection (201212029), and the New York University Institutional Review Board approved the secondary data analysis (IRB-FY2017-213). Participants provided verbal and written consent. This study received a waiver of documentation of consent because all the study data were deidentified.

### Study Recruitment and Survey Administration

**Figure 1:**
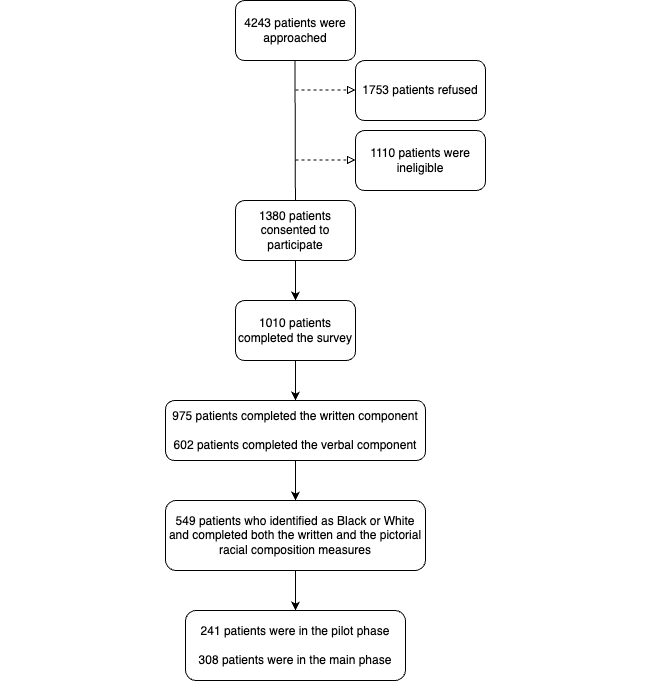
Survey recruitment diagram.

Figure 1 outlines the convenience sampling approach used to recruit study participants. Study recruitment details have been described elsewhere [55,58,61-65], but in brief, trained research assistants (RAs) approached 4243 individuals in the COH waiting room between July 1, 2013, and April 29, 2014. Eligible patients were aged at least 18 years, a COH patient, and English speaking. Of the 4243 individuals, 41.32% (1753/4243) refused to participate, 26.16% (1110/4243) were ineligible, and 32.52% (1380/4243) provided verbal and written consent to participate in the study.

RAs asked the participants to complete a self-administered written questionnaire and a verbally administered survey component. Surveys were administered on different days of the week and at different times of the day. Of the 1380 participants who gave consent, 73.19% (1010/1380) completed the survey. Survey completion was measured as (1) full completion of the written component or (2) three-fourths completion of the written component and full completion of the verbal component. The primary reason for incomplete surveys was inadequate time between the start of the survey and when the clinic was ready to begin the patient evaluation. Survey respondents were similar to the underlying COH primary care clinic patient population concerning sex, age, race, and location in St Louis. The final analytic sample consisted of 549 respondents who identified as non-Hispanic White or Black (including multiracial) and completed both the written and the pictorial measures. The pilot phase included 241 respondents, and the main phase included 308 respondents.

### Measurement Development Stages

Development of the PRCM occurred across the pilot and main phases. From July 1 to August 6, 2013 (pilot phase), the PRCM was self-administered as part of the written survey component. During this phase, RAs also conducted cognitive interviews with 13 participants. From August 13, 2013, to April 30, 2014 (main phase), RAs verbally administered the PRCM to each participant in private COH conference rooms. We refined the PRCM using insights from cognitive interviews and focus groups (data not shown). This refinement leverages mixed methods research and cognitive response interviews, which are important components in developing survey measures, especially among populations with varying levels of health literacy [66].

### Cognitive Interviews

We used cognitive interviews to improve the validity of the PRCM. One advantage of using this technique is the higher potential for accurately assessing the racial composition of social environments over the life course [67]. Another advantage is the systematic identification and reduction of measurement errors during the measurement development stage [68]. Cognitive interview participants were selected from the clinic population. We conducted 13 cognitive interviews and stopped once we reached saturation. We achieved saturation in the data with <10 participants but continued with a few more interviews to ensure we had captured important perspectives.

RAs conducted cognitive interviews with 10 Black participants during the pilot phase. RAs presented 10 sets of pictures to these participants. They asked them to select the image that best described the racial composition of the following social environments: current or most recent workplace, place of worship, high school (HS), HS classroom, junior HS, junior HS classroom, current neighborhood, neighborhood growing up, current block, and block growing up. Participants recommended changes to the PRCM. We report selected recommendations and responses in the Results section.

### Racial Composition Measures

#### Written Measure

The written survey component included an item adapted from the Behavioral Risk Factor Surveillance System [69] and the National Survey of Black Americans [70] to measure racial composition. This item asked participants to identify the racial composition of 10 social environments from their past and present: neighborhoods and blocks (adolescent and current), schools and classrooms (junior high and HS), workplace, and place of worship. The 8 possible responses included: mostly Black, about half Black, some Black, mostly White, about half White, some White, did not live or attend school in the United States (only applicable to school environments), and not applicable. We categorized participants’ responses into mostly Black, half Black, or mostly White. Participants who indicated that they did not experience the environment in the United States or that the environment was not applicable to them were excluded from the analysis for that environment.

#### Pictorial Measure

To construct the PRCM, we used an adapted version of the “neighborhood cards” developed by Krysan and Farley [56]. Figures S1-S10 in Multimedia Appendix 1 show the adapted measures used in the main phase. Different versions of the adapted measure were used during the pilot phase, but they were revised for the main phase based on results from the cognitive interviews and focus groups. RAs presented this pictorial measure to the participants and asked them to select the approximate racial composition of 10 social environments. The 7 possible responses to the pictorial measure included: all Black, 70% Black, 50% Black, 30% Black, 10% Black, all White, and not applicable. As with the written measure, we collapsed the answer choices into the same 3 categories: all Black and 70% Black were categorized as mostly Black, 50% Black and 30% Black were categorized as half Black, and 10% Black and all White were categorized as mostly White. Those who selected not applicable were not included in the analysis for that environment.

### Demographics and Health Literacy

Self-reported demographic characteristics included sex (male or female), race (non-Hispanic White or Black), age, current county (St Louis City, St Louis County, or other), education (less than HS, HS or General Educational Diploma, or more than HS), employment status (currently in the workforce or not), and income (<US $9999, US $10,000-$19,999, or >US $20,000). Health literacy was assessed using the Rapid Estimate of Adult Literacy in Medicine–Revised (REALM-R) [71,72] and the Newest Vital Sign (NVS) [73].

### Analytic Strategy

We calculated several validity metrics by comparing written and PRCMs (Textbox 1). For each social environment and survey phase (pilot and main), we computed positive versus negative pairwise comparisons: (1) mostly Black versus all other categories, (2) half Black versus all other categories, and (3) mostly White versus all other categories. We calculated the following validity metrics for each pairwise comparison: sensitivity, specificity, correct classification rate (CCR), positive predictive value (PPV), negative predictive value (NPV), positive likelihood ratio (LR+), negative likelihood ratio (LR–), false positive rate, and false negative rate. All validity statistics were calculated using SAS and STAT software (version 9.4; SAS Institute Inc).

**Statistical metrics for validating the Pictorial Racial Composition Measure.**

**Metric and description**

- Sensitivity (true positive rate)
  Probability of choosing the positive category (eg, mostly Black) on the pictorial and written measures, given that the positive category was selected on the written measure
- Specificity (true negative rate)
  Probability of choosing the negative category (eg, all other categories) on the pictorial and written measures, given that the negative category was selected on the written measure
- Correct classification rate (accuracy)
  Proportion of participants who identified the same category (positive or negative) on the pictorial and written measures
- Positive predictive value
  Probability of choosing the positive category on the pictorial and written measures, given that the positive category was chosen on the pictorial measure
- Negative predictive value
  Probability of choosing the negative category on the pictorial and written measures, given that the negative category was chosen on the pictorial measure
- Positive likelihood ratio
  Ratio of the true positive rate and the false positive rate
- Negative likelihood ratio
  Ratio of the false negative rate and the true negative rate
- False positive rate (type 1 error)
  Probability of choosing the positive category on the pictorial measure and the negative category on the written measure, given that the negative category was selected on the written measure
- False negative rate (type 2 error)
  Probability of choosing the negative category on the pictorial measure and the positive category on the written measure, given that the positive category was selected on the written measure

## Results

### Cognitive Interviews

Table 1 presents descriptive statistics of the 10 Black cognitive interview participants and their recommended changes to the PRCM. Most of the participants were female (7/10, 70%), aged at least 26 years (9/10, 90%), HS graduates (9/10, 90%), and earning <US $30,000 (9/10, 90%). In addition, the proportions of those with inadequate health literacy differed between the REALM-R and the NVS.

**Table 1:** Characteristics of the 10 Black cognitive interview participants and their recommended changes to the Pictorial Racial Composition Measure.

						Participants, n (%)
**Characteristic**						
	**Sex**					
		Male			3 (30)	
		Female			7 (70)	
	**Age** **(y)**					
		≤25			0 (0)	
		26-35			3 (30)	
		36-49			2 (20)	
		>50			4 (40)	
		Missing			1 (10)	
	**Education**					
		Less than high school			0 (0)	
		High school or General Educational Diploma			5 (50)	
		Greater than high school			4 (40)	
		Missing			1 (10)	
	**Income** **(US $)**					
		<9999			4 (40)	
		10,000-29,999			5 (50)	
		>30,000			0 (0)	
		Missing			1 (10)	
	**Health literacy**					
		**REALM-R^a^**				
			Adequate health literacy	5 (50)		
			Inadequate health literacy	5 (50)		
		**Newest Vital Sign**				
			Adequate health literacy	0 (0)		
			Inadequate health literacy	10 (100)		
**Recommended changes**						
	Workplace				3 (30)	
	Place of worship				1 (10)	
	High school				4 (40)	
	High school classroom				4 (40)	
	Junior high				4 (40)	
	Junior high classroom				4 (40)	
	Current neighborhood				6 (60)	
	Neighborhood growing up				6 (60)	
	Current block				3 (30)	
	Block growing up				4 (40)	

^a^REALM-R: Rapid Estimate of Adult Literacy in Medicine–Revised.

Among the 10 social environments, the initial items for 3 environments had the lowest number of respondents who recommended changes be made: place of worship, current workplace, and current block. These results indicate that respondents experienced the least confusion when seeing those pictures. For example, when asked to select the picture that best described the racial makeup of their place of worship, 1 respondent answered, “it’s asking the race that I worship with, that I go to church with. It’s an all-black church.” This demonstrates that these set of pictures were easy to interpret.

In contrast, participants reported that the past and current neighborhood pictures were the most difficult to interpret. Reasons for this included the indistinguishable color of the houses, the small size of the houses, and the placement of trees. For example, 1 person asked, “Are those supposed to be trees?...It’s kind of confusing.” Another respondent found the pictures unclear, stating, “Now this one is kind of confusing to me as far as these little houses are concerned, because I’m not quite sure if the ones that are painted black mean that black people live there and the ones painted white means white people.” Respondents suggested altering the picture to “make the houses bigger” and “adding people*”* to make it clearer.

### Survey Data: Analytic Sample Characteristics

As shown in Table 2, most of the analytic sample was female (363/549, 66.1%), Black (364/549, 66.3%), and aged >44 years (387/549, 70.5%). Approximately half (257/549, 46.8%) of the participants lived in St Louis City, and 82.1% (451/549) of the participants had at least a HS education. Most participants reported earning <US $20,000 (354/549, 64.4%) and not being in the workforce (408/549, 74.3%). A higher proportion of the sample was categorized as having inadequate health literacy using the NVS than the REALM-R.

**Table 2:** Characteristics of the analytic survey sample.

Characteristic			Overall (n=549), n (%)		Pilot phase (n=241), n (%)	Main phase (n=308), n (%)
**Sex**						
	Male		177 (32.2)		76 (31.5)	101 (32.8)
	Female		363 (66.1)		156 (64.7)	207 (37.2)
	Missing		9 (1.6)		9 (3.7)	0 (0)
**Race**						
	Non-Hispanic White, no multiracial participants		185 (33.7)		80 (33.2)	105 (34.1)
	Black, including multiracial participants		364 (66.3)		161 (66.8)	203 (65.9)
**Age (years)**						
	18-24		10 (1.8)		3 (1.2)	7 (2.3)
	25-34		49 (8.9)		22 (9.1)	27 (8.8)
	35-44		84 (15.3)		43 (17.8)	41 (13.3)
	45-54		175 (31.9)		67 (27.8)	108 (35.1)
	55-64		164 (29.9)		72 (29.9)	92 (29.9)
	>65		48 (8.7)		27 (11.2)	21 (6.8)
	Missing		19 (3.5)		7 (2.9)	12 (3.9)
**Current county**						
	St Louis City		257 (46.8)		123 (51.0)	134 (43.5)
	St Louis County		175 (31.9)		75 (31.1)	100 (32.5)
	Other		90 (16.4)		35 (14.5)	55 (17.9)
	Missing		27 (4.9)		8 (3.3)	19 (6.2)
**Education**						
	Less than high school		82 (14.9)		34 (14.1)	48 (15.6)
	High school or General Educational Diploma		195 (35.5)		79 (32.8)	116 (37.7)
	More than high school		256 (46.6)		116 (48.1)	140 (45.5)
	Missing		16 (2.9)		12 (5.0)	4 (1.3)
**Employment status**						
	Currently in workforce		109 (19.9)		53 (22)	56 (18.2)
	Not currently in workforce		408 (74.3)		179 (74.3)	229 (74.4)
	Missing		32 (5.8)		9 (3.7)	23 (7.5)
**Income (US $)**						
	<9999		227 (41.3)		105 (43.6)	122 (39.6)
	10,000-19,999		127 (23.1)		61 (25.3)	66 (21.4)
	>20,000		137 (25)		57 (23.7)	80 (26)
	Missing		58 (10.6)		18 (7.5)	40 (13)
**Health literacy**						
	**REALM-R^a^**					
		Adequate health literacy	282 (51.4)	116 (48.1)		166 (53.9)
		Inadequate health literacy	231 (42.1)	92 (38.2)		139 (45.1)
		Missing	36 (6.6)	33 (13.7)		3 (1)
	**Newest Vital Sign**					
		Adequate health literacy	191 (34.8)	87 (36.1)		104 (33.8)
		Inadequate health literacy	323 (58.8)	124 (51.5)		199 (64.6)
		Missing	35 (6.4)	30 (12.4)		5 (1.6)

^a^REALM-R: Rapid Estimate of Adult Literacy in Medicine–Revised.

### Pilot Phase

Validity statistics for the pictorial against the written racial composition measure during the pilot phase are summarized in Table 3. Across all 10 social environments, both dichotomizations of *mostly Black versus other categories* and *mostly White versus other categories* exhibited a moderate-to-high sensitivity, specificity, CCR, PPV, and NPV. The LR+ values were >1, and the LR– values were close to 0. The false positive and negative rates were low to moderate. These values indicate that using either the *mostly Black versus other categories* or the *mostly White versus other categories* dichotomizations may provide accurate and reliable measures of racial composition across the 10 social environments.

**Table 3:** Validity statistics for the Pictorial Racial Composition Measure against the written racial composition measure—pilot phase (n=241).

Social environment		Sensitivity	Specificity	CCR^a^	PPV^b^	NPV^c^	LR+^d^	LR–^e^	False positive rate	False negative rate
**Current workplace (n=122)**										
	Mostly Black	55.56	82.56	74.59	57.14	81.61	3.19	0.54	17.44	44.44
	Half Black	42.42	73.03	64.76	36.84	77.38	1.57	0.79	26.97	57.58
	Mostly White	67.92	81.16	75.41	73.47	76.71	3.61	0.40	18.84	32.08
**Current place of worship (n=144)**										
	Mostly Black	86.90	81.67	84.72	86.90	81.67	4.74	0.16	18.33	13.10
	Half Black	44.44	85.71	80.56	30.77	91.53	3.11	0.65	14.29	55.56
	Mostly White	66.67	94.12	86.11	82.35	87.27	11.34	0.35	5.88	33.33
**HS^f^ (n=198)**										
	Mostly Black	86.60	94.06	90.40	93.33	87.96	14.58	0.14	5.94	13.40
	Half Black	62.50	90.36	85.86	55.56	92.59	6.48	0.42	9.64	37.50
	Mostly White	89.86	92.25	91.41	86.11	94.44	11.59	0.11	7.75	10.14
**HS classroom (n=192)**										
	Mostly Black	93.10	91.43	92.19	90.00	94.12	10.86	0.08	8.57	6.90
	Half Black	48.39	94.41	86.98	62.50	90.48	8.66	0.55	5.59	51.61
	Mostly White	90.54	90.68	90.63	85.90	93.86	9.71	0.10	9.32	9.46
**Junior HS (n=172)**										
	Mostly Black	87.50	92.39	90.12	90.91	89.47	11.50	0.14	7.61	12.50
	Half Black	50.00	93.15	86.63	56.52	91.28	7.30	0.54	6.85	50.00
	Mostly White	89.39	87.74	88.37	81.94	93.00	7.29	0.12	12.26	10.61
**Junior HS classroom (n=169)**										
	Mostly Black	94.67	91.49	92.90	89.87	95.56	11.12	0.06	8.51	5.33
	Half Black	52.17	95.89	89.94	66.67	92.72	12.69	0.50	4.11	47.83
	Mostly White	91.55	92.86	92.31	90.28	93.81	12.82	0.09	7.14	8.45
**Current neighborhood (n=193)**										
	Mostly Black	58.95	86.73	73.06	81.16	68.55	4.44	0.47	13.27	41.05
	Half Black	53.33	93.92	84.46	72.73	86.88	8.77	0.50	6.08	46.67
	Mostly White	86.79	67.86	73.05	50.55	93.14	2.70	0.19	32.14	13.21
**Neighborhood growing up (n=192)**										
	Mostly Black	64.71	88.89	76.05	86.84	68.97	5.82	0.40	11.11	35.29
	Half Black	26.67	91.53	86.46	21.05	93.64	3.15	0.80	8.47	73.33
	Mostly White	88.00	73.50	79.17	68.04	90.53	3.32	0.16	26.50	12.00
**Current block (n=194)**										
	Mostly Black	83.67	95.83	89.69	95.35	85.19	20.06	0.17	4.17	16.33
	Half Black	82.86	85.53	85.05	55.77	95.77	5.73	0.20	14.47	17.14
	Mostly White	80.33	94.74	90.21	87.50	91.30	15.27	0.21	5.26	19.67
**Block growing up (n=179)**										
	Mostly Black	83.70	82.76	83.24	83.70	82.76	4.85	0.20	17.24	16.30
	Half Black	11.76	91.98	84.36	13.33	90.85	1.47	0.96	8.02	88.24
	Mostly White	88.57	90.83	89.95	86.11	92.52	9.66	0.13	9.17	11.43

^a^CCR: correct classification rate.

^b^PPV: positive predictive value.

^c^NPV: negative predictive value.

^d^LR+: positive likelihood ratio.

^e^LR–: negative likelihood ratio.

^f^HS: high school.

The *half Black versus other categories* dichotomization yielded a good specificity, CCR, NPV, and false positive rate. However, we observed wide ranges for sensitivity, PPV, and false negative rate. The half Black LR+ ranged from 1 to 13, and the half Black LR– ranged from 0.2 to 1.0. These findings suggest half Black dichotomization may be less accurate and reliable than the mostly Black and mostly White dichotomizations when measuring the racial composition of the 10 social environments.

### Main Phase

Table 4 compares the validity statistics for the pictorial against the written racial composition measure during the main phase. Across all 10 social environments, the dichotomizations of mostly Black versus other categories and mostly White versus other categories yielded a moderate-to-high sensitivity, specificity, CCR, PPV, and NPV. The LR+ values were >1, and the LR– values were close to 0. These results were similar to the pilot phase results. However, the false positive and negative rates in the main phase slightly differed from those in the pilot phase. Overall, these findings further support that using either the mostly Black versus other categories or the mostly White versus other categories dichotomizations may provide accurate and reliable measures of racial composition across the 10 social environments.

The specificity, CCR, NPV, LR+, and LR– were relatively good for the *half Black versus other categories* dichotomization. However, this dichotomization demonstrated poor validity regarding its sensitivity, PPV, and false negative rate. These values suggest that the *half Black versus other categories* dichotomization may not be as accurate and reliable as the *mostly Black versus other categories* and *mostly White versus other categories* dichotomizations.

**Table 4:** Validity statistics for the Pictorial Racial Composition Measure against the written racial composition measure—main phase (n=308).

Social environment		Sensitivity	Specificity	CCR^a^	PPV^b^	NPV^c^	LR+^d^	LR–^e^	False positive rate	False negative rate
**Current workplace (n=190)**										
	Mostly Black	54.35	81.25	74.74	48.08	84.78	2.90	0.56	18.75	45.65
	Half Black	42.86	73.88	64.74	40.68	75.57	1.64	0.77	26.12	57.14
	Mostly White	65.91	79.41	73.16	73.42	72.97	3.20	0.43	20.59	34.09
**Current place of worship (n=222)**										
	Mostly Black	83.90	79.81	81.98	82.50	81.37	4.16	0.20	20.19	16.10
	Half Black	45.24	85.00	77.48	41.30	86.93	3.02	0.64	15.00	54.76
	Mostly White	72.58	93.13	87.39	80.36	89.76	10.56	0.29	6.87	27.42
**HS^f^ (n=281)**										
	Mostly Black	78.69	84.28	81.85	79.30	83.75	5.01	0.25	15.72	21.31
	Half Black	52.94	81.69	74.73	48.00	84.47	2.89	0.58	18.31	47.06
	Mostly White	68.13	87.89	81.49	72.94	85.20	5.63	0.36	12.11	31.87
**HS classroom (n=276)**										
	Mostly Black	88.03	74.84	80.44	72.03	89.47	3.50	0.16	25.16	11.97
	Half Black	33.90	88.48	76.82	44.44	83.12	2.94	0.75	11.52	66.10
	Mostly White	75.00	92.61	86.23	85.23	86.70	10.15	0.27	7.39	25.00
**Junior HS (n=260)**										
	Mostly Black	83.33	82.14	82.69	80.00	85.19	4.67	0.20	17.86	16.67
	Half Black	38.46	86.06	76.54	40.82	84.83	2.76	0.72	13.94	61.54
	Mostly White	73.86	87.79	83.08	75.58	86.78	6.05	0.30	12.21	26.14
**Junior HS classroom (n=260)**										
	Mostly Black	91.96	80.41	85.39	78.03	92.97	4.69	0.10	19.59	8.04
	Half Black	44.44	90.29	80.77	54.55	86.11	4.58	0.62	9.71	55.56
	Mostly White	75.83	92.17	86.16	84.52	86.93	9.68	0.26	7.83	24.17
**Current neighborhood (n=283)**										
	Mostly Black	73.13	85.23	79.51	81.67	77.91	4.95	0.32	14.77	26.87
	Half Black	47.37	81.42	74.56	39.13	85.98	2.55	0.65	18.58	52.63
	Mostly White	73.91	86.39	82.33	72.34	87.30	5.43	0.30	13.61	26.09
**Neighborhood growing up (n=276)**										
	Mostly Black	89.13	88.41	88.77	88.49	89.05	7.69	0.12	11.59	10.87
	Half Black	42.42	88.07	82.61	32.56	91.85	3.56	0.65	11.93	57.58
	Mostly White	75.24	91.23	85.14	84.04	85.71	8.58	0.27	8.77	24.76
**Current block (n=277)**										
	Mostly Black	81.89	82.67	82.32	80.00	84.35	4.73	0.22	17.33	18.11
	Half Black	43.33	86.18	76.90	46.43	84.62	3.14	0.66	13.82	56.67
	Mostly White	74.44	87.17	83.03	73.63	87.63	5.80	0.29	12.83	25.56
**Block growing up (n=277)**										
	Mostly Black	83.67	81.54	82.67	83.67	81.54	4.53	0.20	18.46	16.33
	Half Black	29.03	89.43	82.67	25.71	90.91	2.75	0.79	10.57	70.97
	Mostly White	79.80	91.01	87.00	83.16	89.01	8.88	0.22	8.99	20.20

^a^CCR: correct classification rate.

^b^PPV: positive predictive value.

^c^NPV: negative predictive value.

^d^LR+: positive likelihood ratio.

^e^LR–: negative likelihood ratio.

^f^HS: high school.

## Discussion

### Principal Findings

We developed and validated the PRCM, a measure of the racial composition of social environments over the life course. Pilot and main phase results across all 10 social environments showed that the mostly Black and mostly White dichotomizations exhibited moderate-to-high scores for sensitivity, specificity, CCR, PPV, and NPV. These dichotomizations also yielded acceptable values for LR+ and LR– and false positive and negative rates. These findings support that the mostly Black and mostly White dichotomizations may provide accurate and reliable measures of racial composition across the 10 social environments.

The PRCM provides an added window into understanding how racial segregation has impacted minoritized communities and can be used to investigate interpersonal, institutional, structural, and systemic racism [74,75]. Although further empirical research is needed from a generalizable population, the mostly Black and mostly White dichotomization findings provide preliminary evidence that an optimal cut-off point exists for the categorization of segregation in social environments. We validated a pictorial measure in a population composed mainly of those with low health literacy according to the NVS. Thus, we provide a novel and accessible resource for clinical researchers to measure the racial composition of their patient’s social environments. Analyses using the PRCM may lead to a more comprehensive understanding of how segregated social environments over the life course affect health outcomes.

The mostly Black and mostly White dichotomizations performed better than the half Black dichotomization, possibly because the older study population (387/549, 70.5% aged at least 45 years) may have been more likely to remember predominantly Black or White social environments compared to 50% Black environments. In addition, we grouped 50% Black and 30% Black together for the half Black dichotomization. It may be that 30% Black is too far from 50%, potentially contributing to the poor validation of the half Black dichotomization. Overall, these results were similar to those of sensitivity analyses stratified by race (data not shown). There was also a smaller number of participants reporting half Black environments, providing limited data upon which to validate this level.

All explored social environments exhibited good validity metrics for mostly Black and mostly White dichotomizations. However, neighborhood growing up, current neighborhood, and current workplace tended to perform the lowest. This could stem from the images on the pictorial cards for those environments. The neighborhood cards showed houses, which might not resonate with participants who lived in alternative housing arrangements such as apartments or mobile homes. Similarly, the workplace pictorial cards displayed office desks with computers. It is possible that some participants may have never worked in an office setting.

### Comparison With Prior Work

Other approaches used to measure racial segregation include the Index of Concentration at the Extremes [19,20], the dissimilarity index [21,22], the isolation index [23,24], the interaction index [23,25], the location quotient of residential segregation [26,27], and the Getis-Ord Gi* statistic [28]. The Index of Concentration at the Extremes assesses the degree to which individuals in an area are privileged [19,20]. The dissimilarity index is the proportion of individuals considered marginalized needing to relocate to create an equal racial and ethnic distribution within the population [21,22]. The isolation index estimates the spatial proximity between individuals of the same racial category living in the same areas [23,24]. The interaction index estimates the spatial proximity between individuals of different racial categories residing in the same areas [23,25]. The location quotient of residential segregation compares the racial and ethnic distribution of a smaller geographic region to that of a larger geographic region [26,27]. The Getis-Ord Gi* statistic measures each neighborhood’s racial composition and compares it to surrounding neighborhoods [28].

Despite the advantages of these census-based approaches, none can measure the potentially varying racial compositions of multiple social environments across the life course. These census-based approaches may capture regions that do not match what participants consider as their neighborhood [16,29]. An example is a Black participant living in a census region but attending a predominantly White school or a racially integrated church in another census region. Another shortcoming is that these census-based measurements have yet to be validated in low health literacy populations. Finally, repeated assessments of racial segregation over time for each study participant are prone to missing data and may require data imputation techniques (eg, last observation carried forward and multiple imputation) [76].

The validity of the mostly Black and mostly White dichotomizations of the PRCM demonstrates that this measure can assess the racial composition of social environments spanning adolescence and adulthood without depending on census data. Moreover, the PRCM allows the participant to self-report the racial composition of their social environments, negating the potential misclassification of what participants consider their neighborhood. Another advantage of the PRCM is that it can be administered at a single study visit, reducing the need to impute data for responses over time. In addition, because it is picture based, the PRCM may help diversify survey question types and reduce respondent fatigue. Finally, the PRCM was validated using a mixed methods approach in a medically underserved patient population with varied health literacy levels, including those with limited health literacy. However, convenience sampling from a single midwestern primary care clinic may limit generalizability, creating the need to validate the PRCM in other populations.

While we have presented cognitive interview data from Black participants used to inform the PRCM, we also collected data from a few non-Hispanic White participants. We recruited a subset of participants from the COH patient population that was approximately 30% White. However, we reached saturation early in the cognitive interviews and decided to stop recruitment with a lower proportion of White cognitive interview participants. Data were analyzed stratified by race, and the interview data from White participants were concordant with the themes observed with data from Black participants. As the PRCM is refined or being adapted for other studies, additional cognitive interviewing would be helpful. In addition, there were some modifications based on the cognitive interviews to the instrument and the mode of administration from the pilot phase to the main phase. While these changes were made to improve the measure, some of these changes may not have had the intended results and will require additional refinement in future research. We do not have any empirical data to support this, but we hypothesize that changing the mode of administration from written self-administered in the pilot phase to verbally administered by RAs in the main phase may have impacted the validity, with participants more likely to report socially desirable answers to the RAs.

Social, clinical, and public health scientists can use PRCM data in a variety of ways in regression models. One example is creating a composite score, summing the number of mostly Black (or mostly White) social environments a study participant reported, and estimating the effect of reporting an additional mostly Black or mostly White environment on the outcome of interest. Another way researchers can use the PRCM data is to calculate the number of discordant matches between the racial composition of each social environment and the study participant’s race. A third way is to administer the PRCM to children, their parents, and grandparents, assessing the potential generational effects of racial segregation. We encourage scientists to use color images (print or digital) when administering the PRCM.

Racial residential integration is a subject of increasing interest among social scientists [77-79]. A systematic review of racial residential integration spanning 60 years yielded 3 main findings [78]. First, the concept of integration has recently focused on racial composition, shifting away from racial and social integration [78]. Second, the measurement methods for integration have changed over time [78]. Third, racial residential integration has evolved from the Black-White dichotomy to including a broader range of races and ethnicities (eg, Asian and multiethnic) [78]. The authors of this review also note that inconsistent census cutoffs hinder the comparison of integration effects across studies [78]. To unify the study of racial residential integration, the authors recommend developing “a multidimensional theoretical framework for residential integration that includes both racial composition and social interactions” [78]. The PRCM contributes to this goal by helping us understand the range of racial segregation and integration, especially in residential, educational, workplace, and religious contexts.

### Limitations and Future Work

Although we developed a novel measure of racial segregation, we must recognize the limitations of the PRCM and this study. This work was conducted in St Louis, Missouri, where the majority of the population identify as non-Hispanic White alone or Black alone. Given the limited diversity of the community from which the sample was taken, the response options were meant to represent Black and White neighborhoods. This Black and White comparison is a major limitation and requires validation in other populations outside of St Louis before adaptation and use.

The PRCM includes pictures of non-Hispanic White and Black social environments. Thus, it cannot measure compositions of other races and ethnicities, such as Asian or American Indian, limiting its capacity to capture the full racial and ethnic composition of social environments. While this is appropriate for the context of this study (St Louis, Missouri), extensions of the PRCM beyond St Louis, Missouri, or those communities with similar racial compositions should incorporate other racial and ethnic identities to enhance the inclusivity of this measure.

The PRCM is limited in its ability to evaluate segregated social environment experiences in other groups considered historically marginalized (eg, Hispanic and multiracial). Recent research shows that the upward mobility of Hispanic people is more similar to that of individuals racialized as White than of those racialized as Black [80]. Future research should extend the PRCM scope to be adapted for use in multiracial populations beyond those primarily composed of individuals racialized as Black or White.

The binary (White vs Black) measurement limits the PRCM’s ability to measure racially integrated social environments. The PRCM is a subjective measure susceptible to recall bias and misclassification. However, the validity results from this study suggest that bias and misclassification were reduced. We also think that the pictures help people remember each social environment. From the interview transcripts, one participant stated as follows: “It reminds me of my neighborhood. Honestly there were no whites around, period. None*.*” Another participant noted the following: “I am having to remember to relate it back to here (taps something) and I’m looking at it now...” Future studies should include empirical data on whether the pictures help participants remember.

The PRCM is a retrospective cross-sectional measure, which cannot infer causality on an outcome. We lacked information on the zip codes where participants grew up, which prevented us from conducting historical analyses. This highlights an area for future research to support longitudinal studies. Convenience sampling was used to recruit study participants. To circumvent sampling error and bias, RAs recruited participants at different times of the day and on different weekdays. We also acknowledge that using 2 subjective methods has its limitations, such as potential same source bias, telescoping effects, and memory distortions. However, standard validation approaches state that new metrics should be compared against the current gold standard, and in this case, the written measure is the current gold standard for subjective racial compositions across social environments. Moreover, people’s perceptions of their environments are also important to measure in addition to objective data, as perceptions may affect some outcomes.

Another limitation was combining Black and multiracial participants into 1 group. Given the small number of multiracial participants, we decided to include them in the analysis instead of excluding them. Most (7/10, 70%) of the multiracial participants were in the pilot sample, with only 3 (30%) in the main phase sample. The multiracial sample was too small to make inferences as an individual group or to create any real bias among the group of Black respondents. In future research, it will be important to validate the measure among individuals with other racial identities. Finally, the PRCM lacks the ability to assess social interactions between races.

We examined the current and past racial compositions of neighborhoods and blocks to obtain a life course examination of residential contexts over time. Future work could examine if adding additional key time points and being specific about ages would improve the life course measurement. Furthermore, we developed a measure that could be used in a sample with varying levels of health literacy, particularly among those with limited health literacy.

Many of the stratified analyses by the NVS and REALM-R showed that no statistically significant differences were found when comparing the concordance of the pictorial and written measures to objective census-based measures of the racial composition of participants’ current neighborhood and HS (Multimedia Appendix 2). However, NVS analyses yielded substantial differences in concordance between the pictorial measure and the objective census-based measure of participants’ current neighborhood racial composition (Multimedia Appendix 2). There are consistent differences in measures of health literacy across the literature [61,81,82]. This is because different measures capture different aspects of health literacy [61,81,82]. In particular, the NVS is a measure that includes numeracy, while the REALM-R is focused on written words [71-73]. We included both measures in our study to capture these differences.

Our selection of patients from the COH primary care clinic was purposeful in that measures are rarely validated among medically underserved populations with varying levels of health literacy. Using the principles of universal design, we believe a measure that is valid and reliable in this population is likely valid among the general US population, but further work is needed to obtain validation metrics in other populations. For example, patients seeking care in a safety net primary care setting may differ from the larger population. Thus, further work is needed to validate the PRCM for use in community contexts.

### Conclusions

Using mixed methods of cognitive interviews (qualitative) and survey (quantitative) data, we developed and validated the PRCM. This pictorial measure increases our ability to examine racial composition in multiple environments across the life course among medically underserved populations. The PRCM can serve as a uniform measure of racial segregation used across disciplines. It can also be used with other measures to inform equitable intervention and prevention efforts, thus improving the lives of those impacted by structural racism.

## Appendix

Multimedia Appendix 1 Pictorial measures.

Multimedia Appendix 2 Additional descriptive statistics and agreement results.

## Abbreviations

CCR: correct classification rate
COH: Center for Outpatient Health
HS: high school
LR–: negative likelihood ratio
LR+: positive likelihood ratio
NPV: negative predictive value
NVS: Newest Vital Sign
PNRC: perceived neighborhood racial composition
PPV: positive predictive value
PRCM: Pictorial Racial Composition Measure
RA: research assistant
REALM-R: Rapid Estimate of Adult Literacy in Medicine–Revised
