# Supplementary material for: Racial Composition of Social Environments Over the Life Course Using the Pictorial Racial Composition Measure: Development and Validation Study
Source: JMIR Public Health Surveill. 2024 Aug 8;10:e55461. doi: 10.2196/55461 (PMC11342016; doi:10.2196/55461)

Figure S1. Please indicate which picture best describes the racial make-up of **your current/most recent workplace** in the figure below.

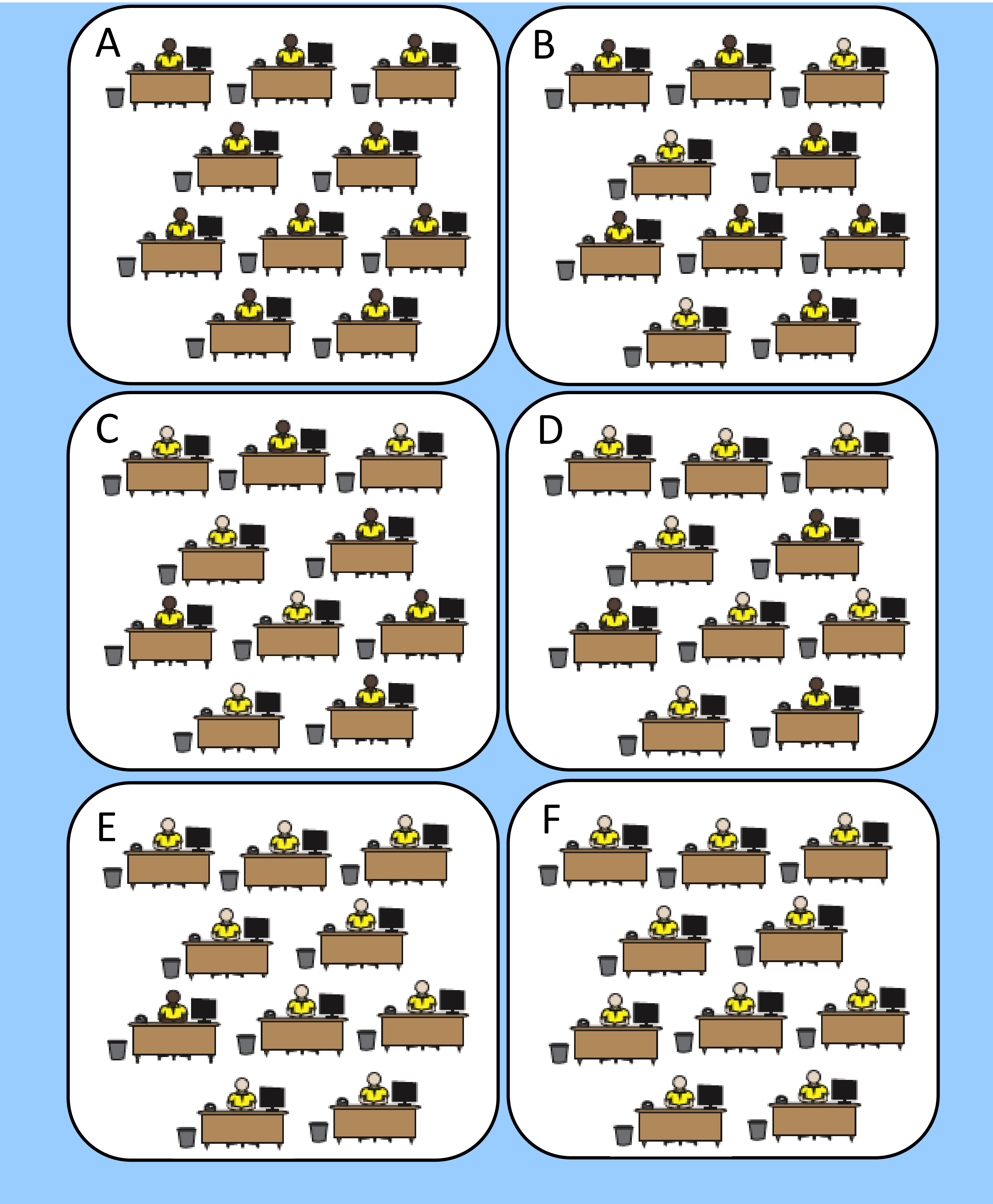

Figure S2. Please indicate which picture best describes the racial make-up of **your <u>place of worship</u>** in the figure below.

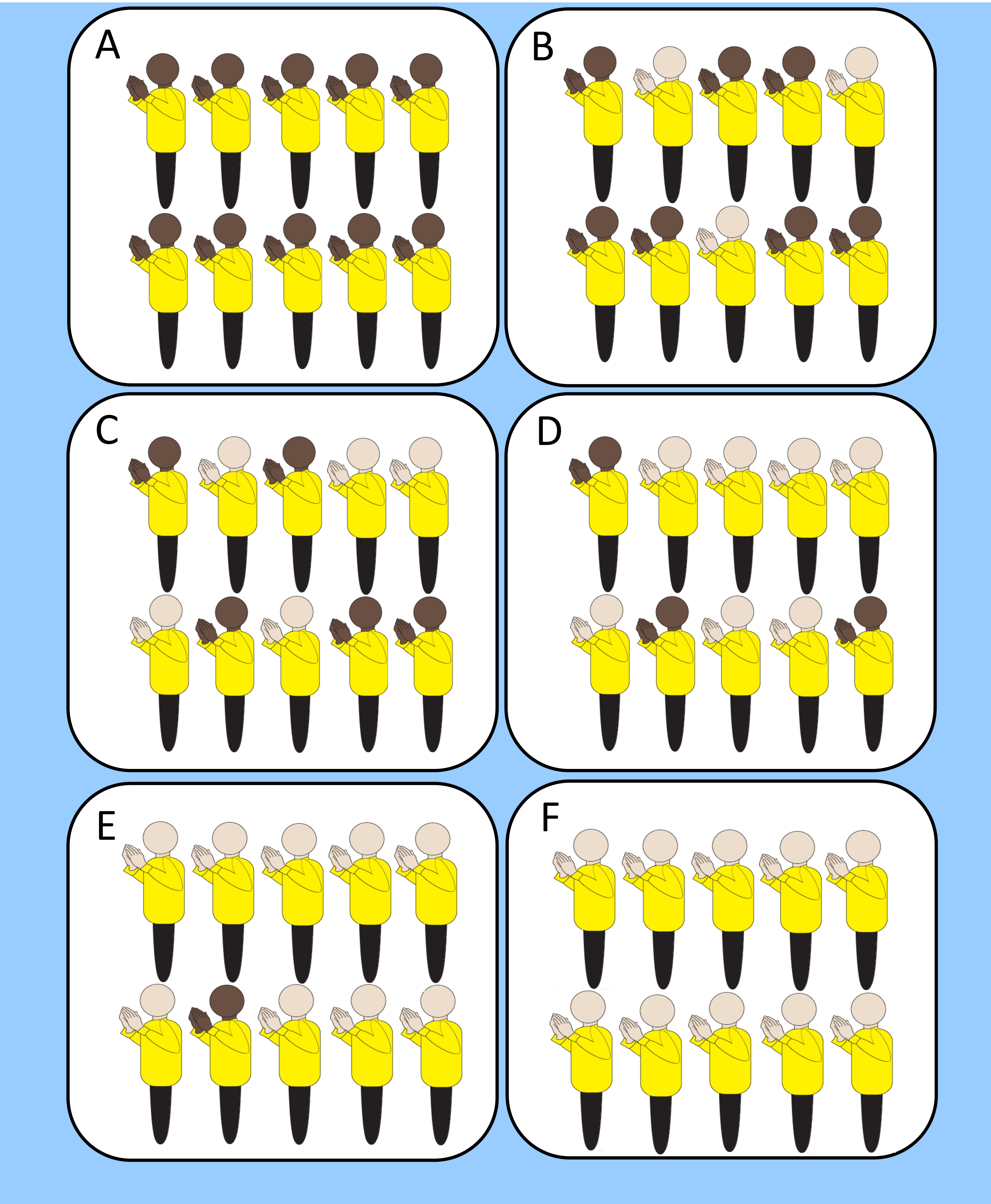

Figure S3. Please indicate which picture best describes the racial make-up of **your <u>high school</u>** in the figure below.

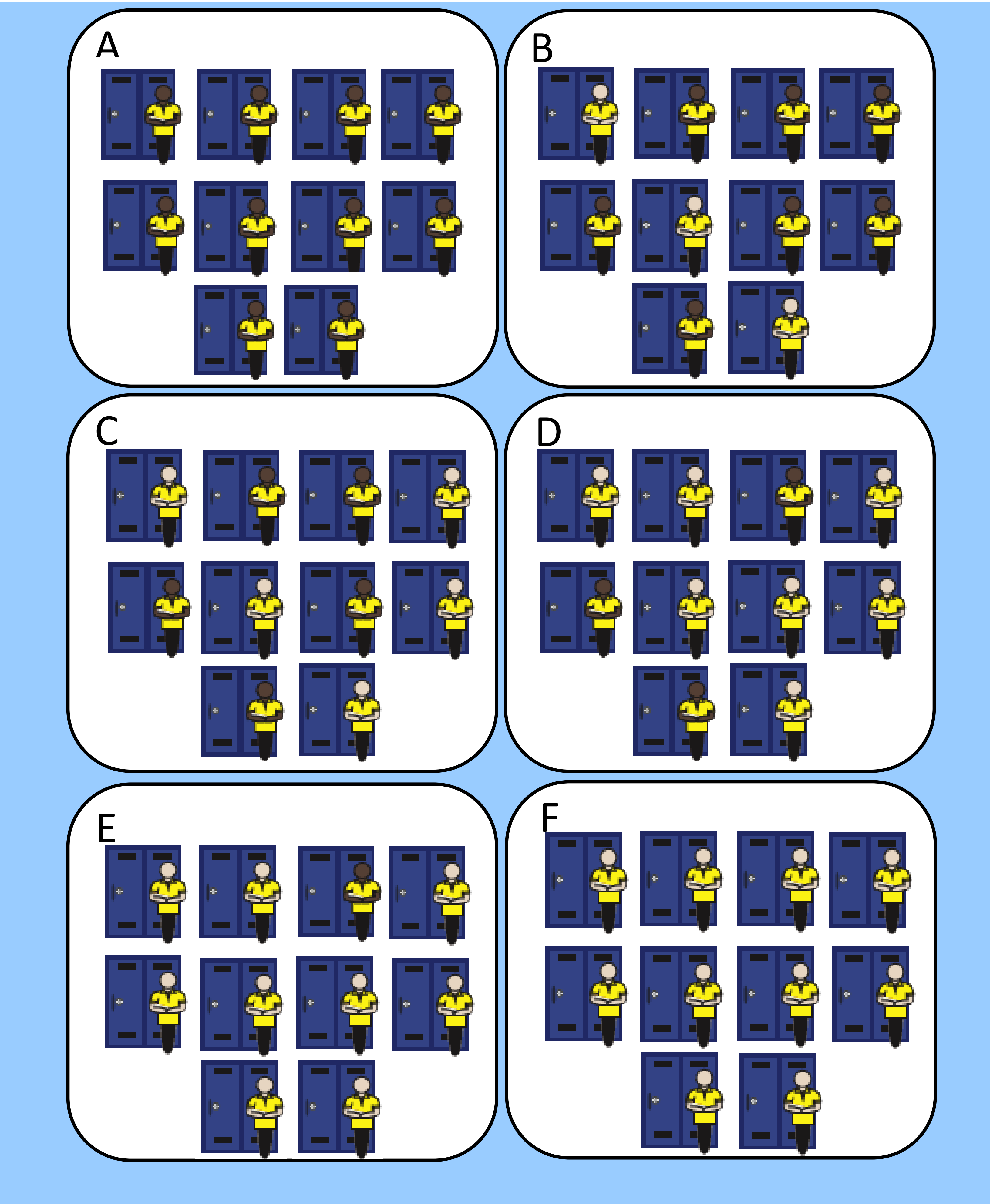

Figure S4. Please indicate which picture best describes the racial make-up of **your <u>high school classroom</u>** in the figure below.

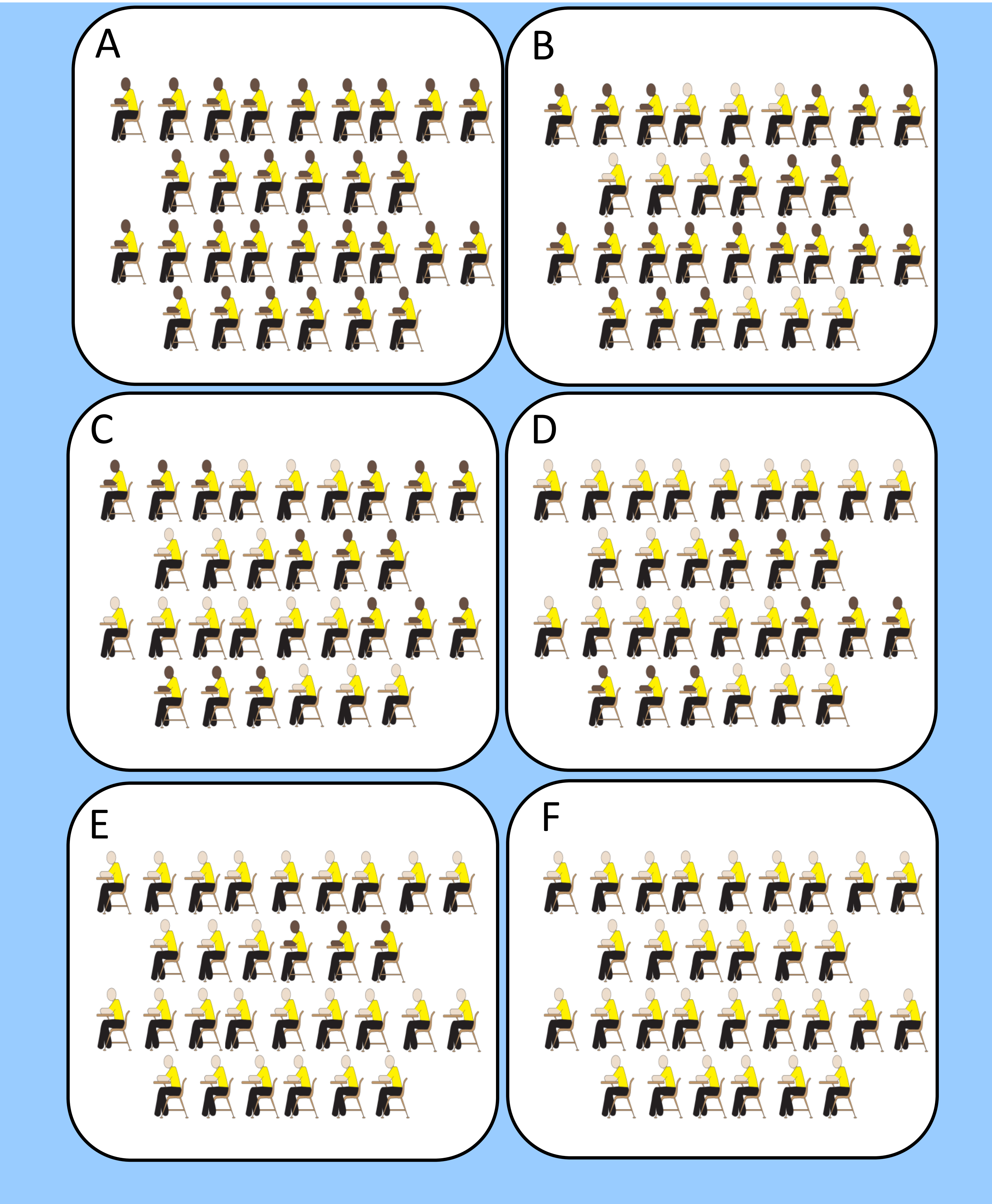

Figure S5. Please indicate which picture best describes the racial make-up of **your junior high school** in the figure below.

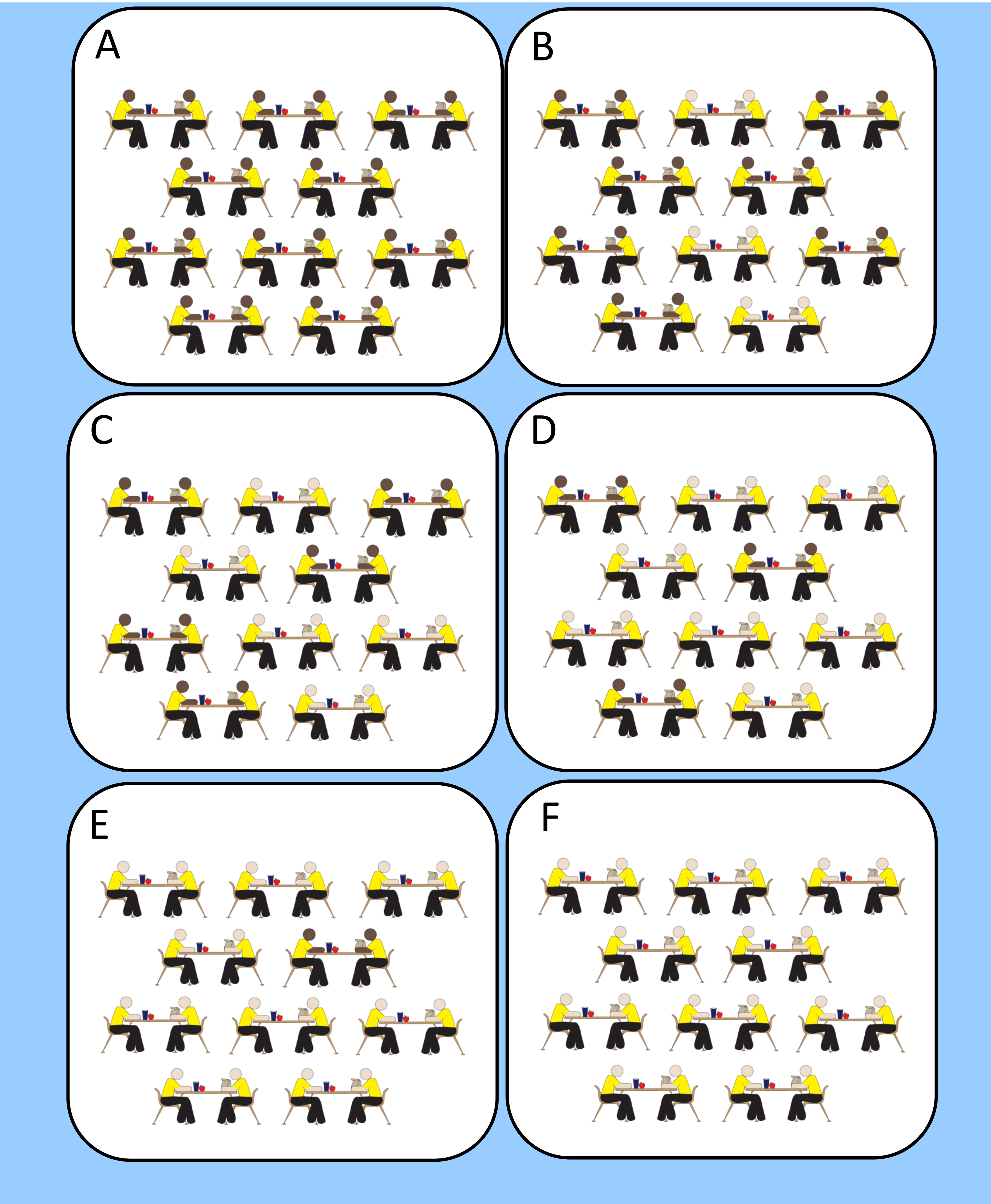

Figure S6. Please indicate which picture best describes the racial make-up of **your <u>junior high school</u>** classroom in the figure below.

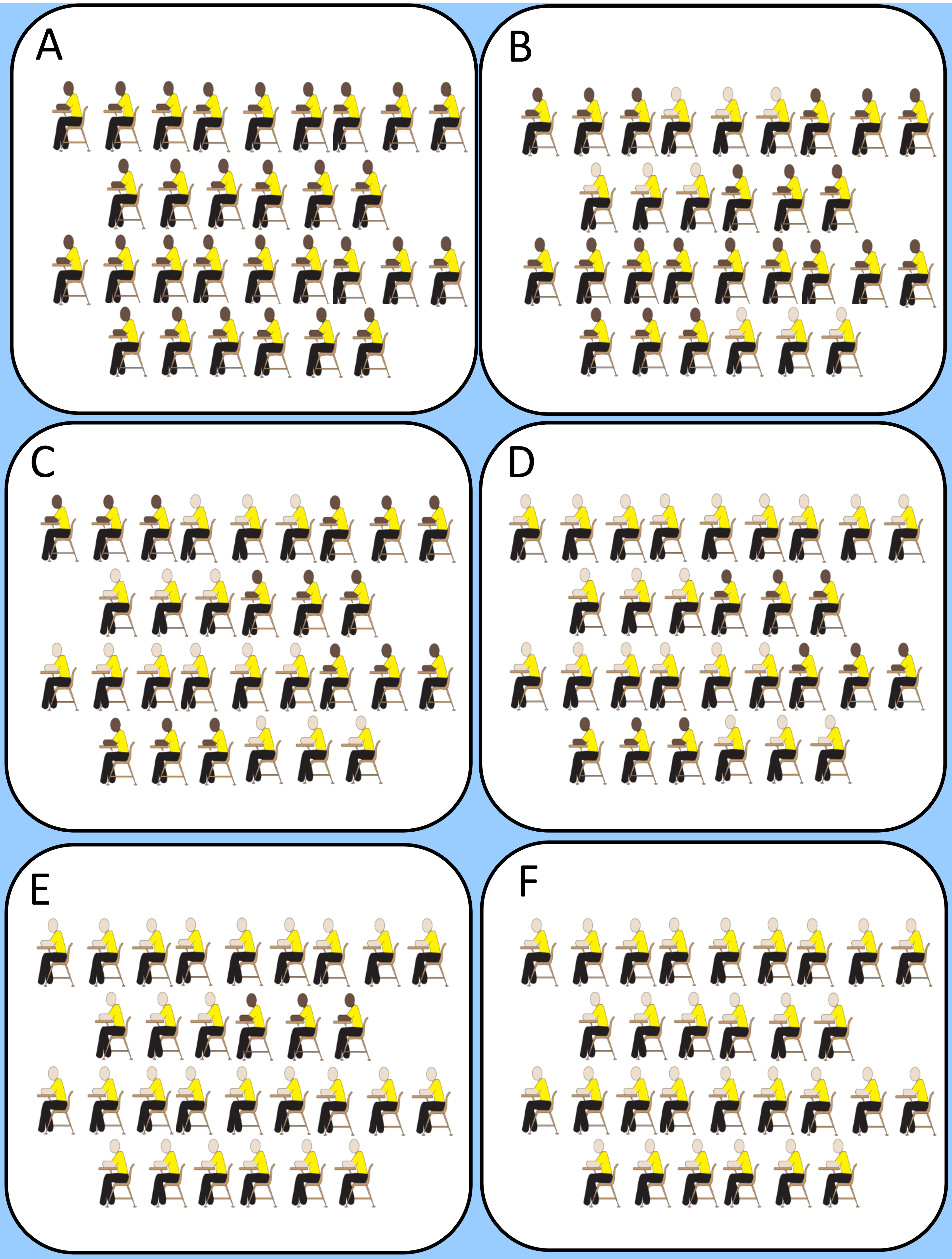

Figure S7. Please indicate which picture best describes the racial make-up of **your <u>current neighborhood</u>** in the figure below.

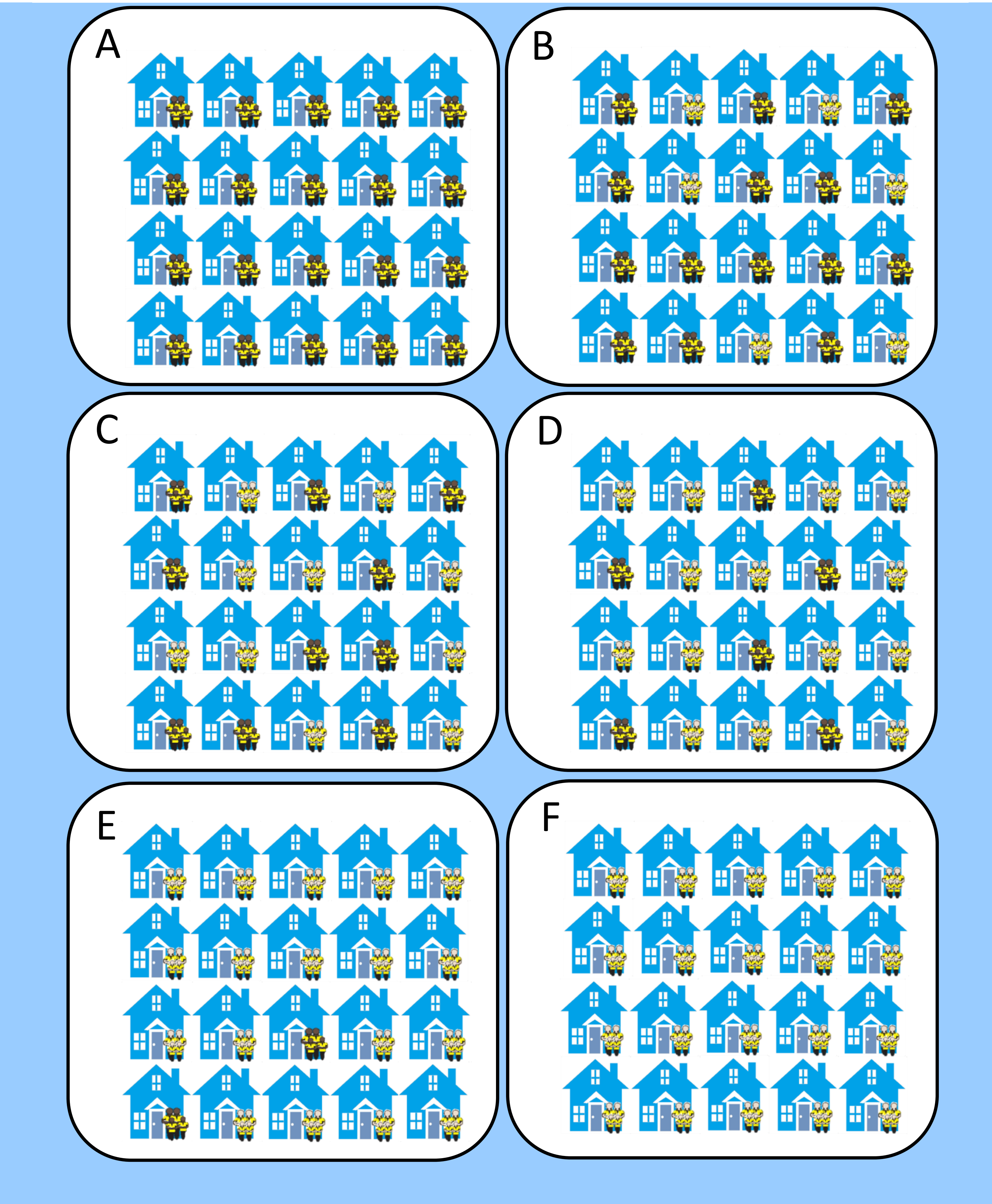

Figure S8. Please indicate which picture best describes the racial make-up of **your <u>neighborhood growing up</u>** in the figure below.

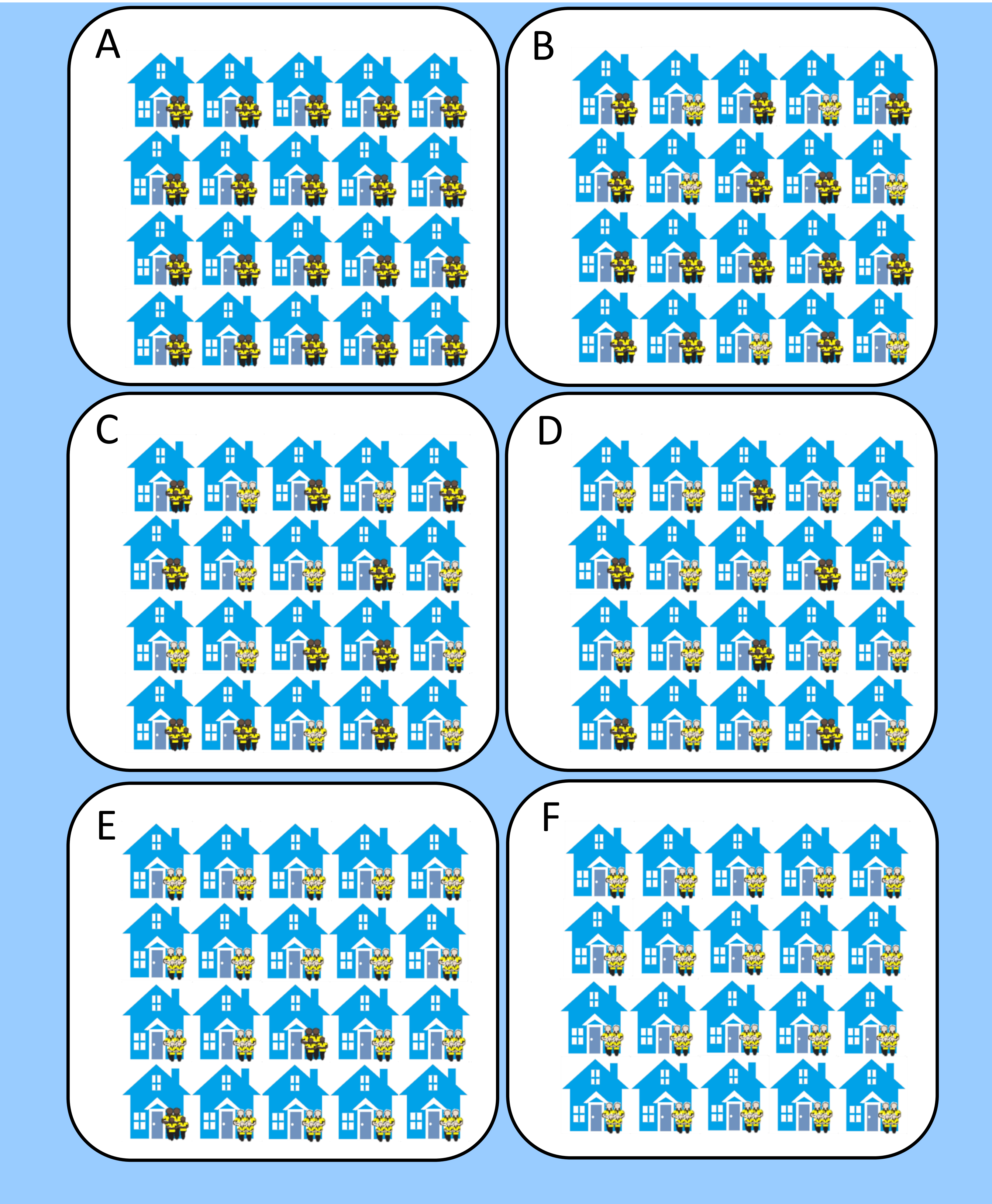

Figure S9. Please indicate which picture best describes the racial make-up of **your** **<u>current block</u>** in the figure below.

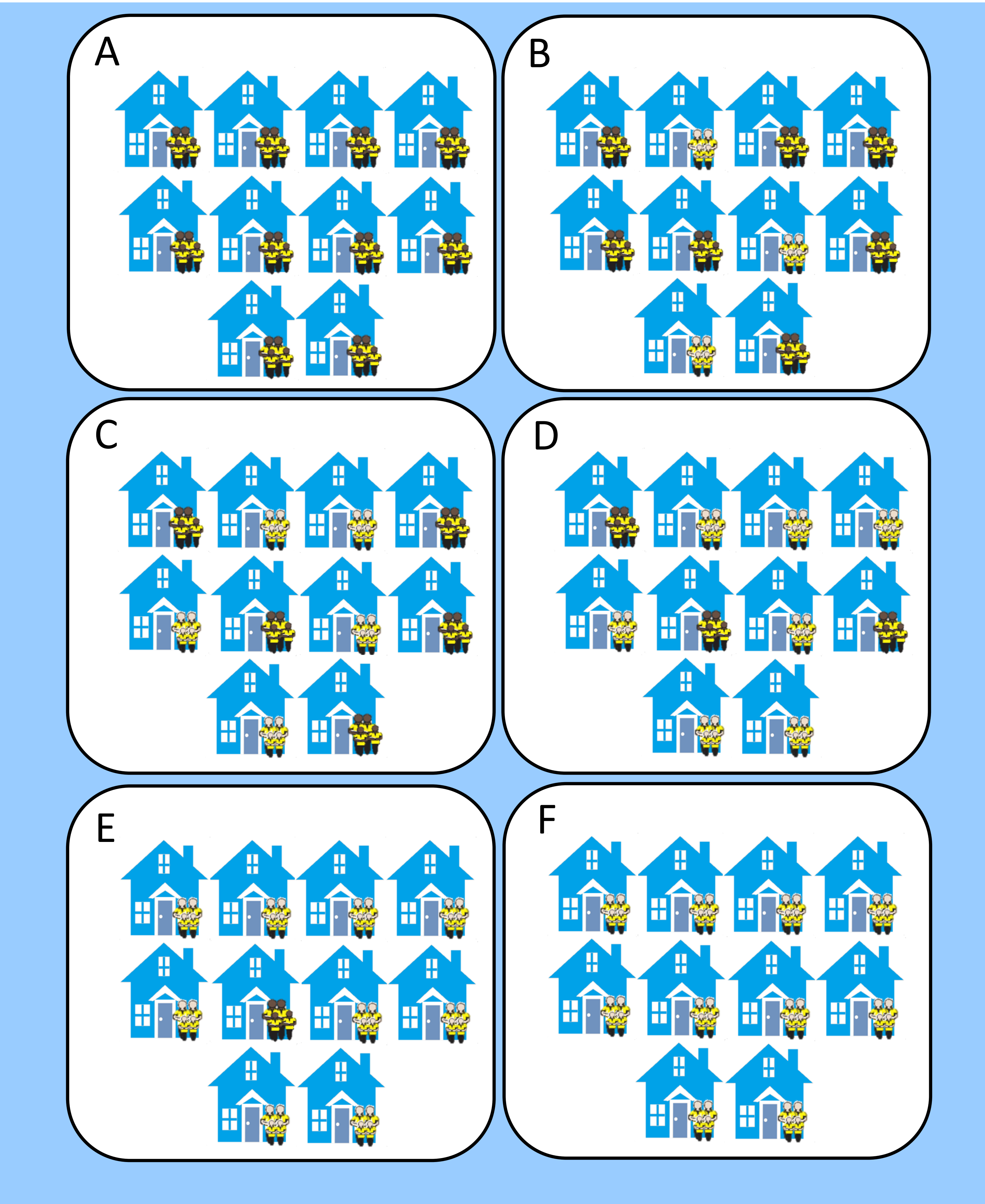

Figure S10. Please indicate which picture best describes the racial make-up of **your <u>block growing up</u>** in the figure below.

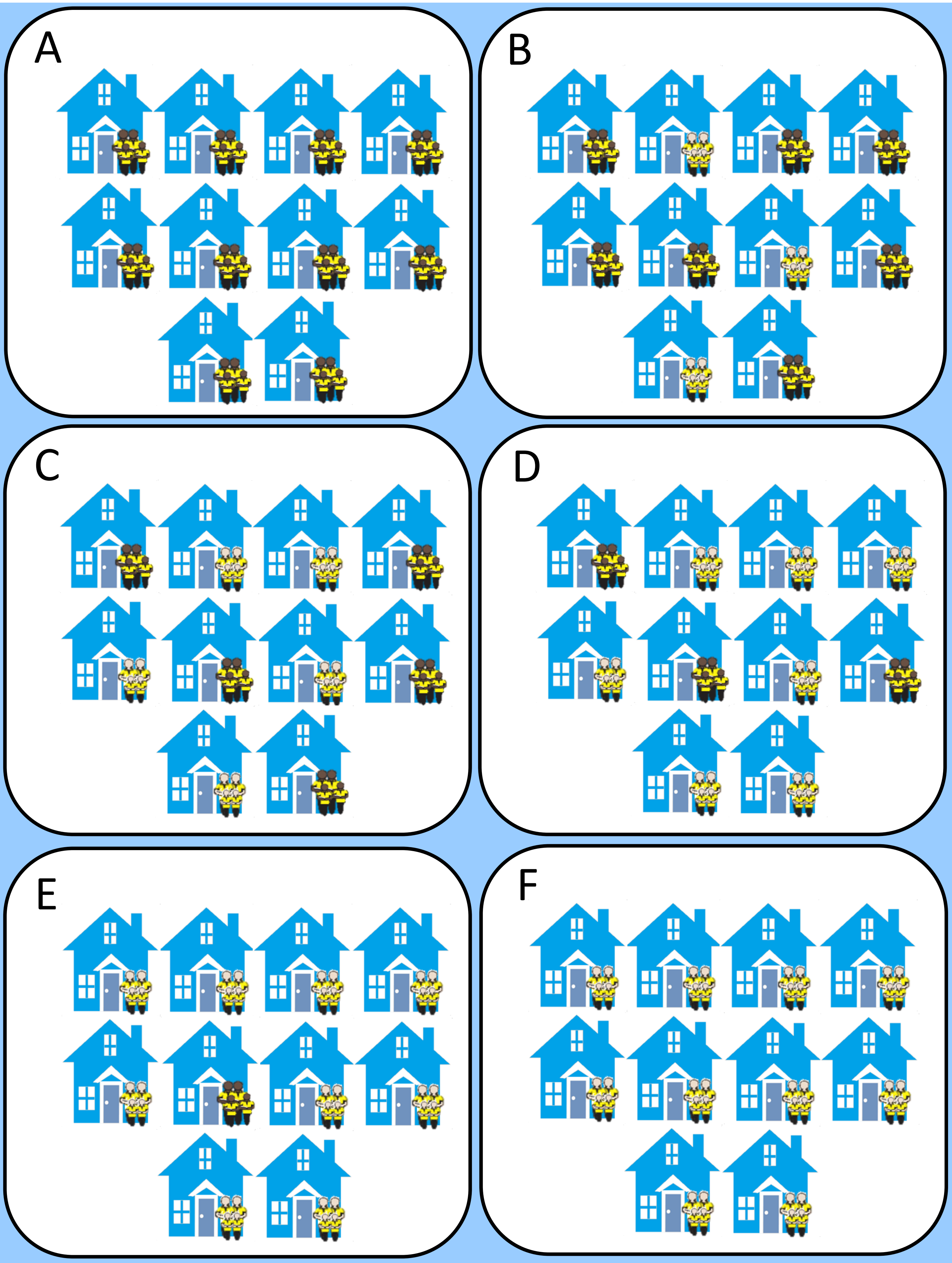

Supplement: Multimedia Appendix 1 [file publichealth_v10i1e55461_app1.pdf]
