# Supplementary material for: Racial Composition of Social Environments Over the Life Course Using the Pictorial Racial Composition Measure: Development and Validation Study
Source: JMIR Public Health Surveill. 2024 Aug 8;10:e55461. doi: 10.2196/55461 (PMC11342016; doi:10.2196/55461)
Supplement: Multimedia Appendix 2 [file publichealth_v10i1e55461_app2.docx]

Supplemental Material for *“Racial Composition of Social Environments Over the Life Course Using the Pictorial Racial Composition Measure: Development and Validation Study”*

Descriptive Statistics for Objective Racial Composition (Percent Black)

by Selection on Pictorial Measure

| Table S1. Descriptive statistics for current neighborhood percent black by pictorial selection | | | | | |
| --- | --- | --- | --- | --- | --- |
| **Selection** | **Mean** | **SD** | **Median** | **Minimum** | **Maximum** |
| 100% Black | 80.97 | 20.96 | 88.48 | 1.78 | 98.63 |
| 70% Black | 66.00 | 27.68 | 72.70 | 1.45 | 98.63 |
| 50% Black | 49.96 | 24.53 | 43.22 | 1.78 | 98.63 |
| 30% Black | 48.84 | 31.23 | 40.88 | 0.71 | 97.32 |
| 10% Black | 34.86 | 29.78 | 29.74 | 0.08 | 96.38 |
| 0% Black | 35.53 | 37.09 | 10.94 | 0.08 | 97.32 |

| Table S2. Descriptive statistics for high school percent black by pictorial selection | | | | | |
| --- | --- | --- | --- | --- | --- |
| **Selection** | **Mean** | **SD** | **Median** | **Minimum** | **Maximum** |
| 100% Black | 61.81 | 32.05 | 65.23 | 1.64 | 99.07 |
| 70% Black | 48.62 | 34.92 | 59.43 | 0.65 | 99.07 |
| 50% Black | 32.49 | 32.02 | 21.27 | 0.49 | 99.07 |
| 30% Black | 13.10 | 22.38 | 1.93 | 0 | 97.49 |
| 10% Black | 9.39 | 17.02 | 1.20 | 0.06 | 52.11 |
| 0% Black | 9.60 | 20.32 | 2.13 | 0.04 | 59.43 |

Measures of Agreement between Categorized Percent Black

and Selection on Pictorial Measure

| Table S3. Measures of agreement for categorized percent black and pictorial selection | | | |
| --- | --- | --- | --- |
| **Statistic** | **Value** | **ASE** | **p-value** |
| Neighborhood |  |  |  |
| Spearman Correlation | 0.5114 | 0.0370 | <.0001 |
| Simple Kappa | 0.1725 | 0.0231 | <.0001 |
| Weighted Kappa | 0.3671 | 0.0280 |  |
| High School |  |  |  |
| Spearman Correlation | 0.3109 | 0.0371 | <.0001 |
| Simple Kappa | 0.0470 | 0.0133 | 0.0003 |
| Weighted Kappa | 0.1317 | 0.0161 |  |

Tables S4 and S5 shows whether the self-reported racial composition from selection on the pictorial measure is concordant with the objective data, an underestimate, or an overestimate. Table S4 looks at the current neighborhood, and Table S5 looks at high school composition.

| Table S4. Pictorial measure results by race for current neighborhood | | | | | | | | |
| --- | --- | --- | --- | --- | --- | --- | --- | --- |
|  | **Overall (n=471)** | | **Black (n=306)** | | **White (n=165)** | |  |  |
| **Relationship** | **N** | **%** | **N** | **%** | **N** | **%** | **Χ^2^** | **p-value** |
| Concordance | 145 | 30.79% | 100 | 32.68% | 45 | 27.27% | 32.66 | <0.0001 |
| Underestimate | 135 | 28.66% | 109 | 35.62% | 26 | 15.76% |  |  |
| Overestimate | 191 | 40.55% | 97 | 31.70% | 94 | 56.97% |  |  |

| Table S5. Pictorial measure results by race for high school | | | | | | | | |
| --- | --- | --- | --- | --- | --- | --- | --- | --- |
|  | **Overall (n=477)** | | **Black (n=310)** | | **White (n=167)** | |  |  |
| **Relationship** | **N** | **%** | **N** | **%)** | **N** | **%** | **Χ^2^** | **p-value** |
| Concordance | 77 | 16.14% | 42 | 13.55%) | 35 | 20.96% | 10.03 | 0.0066 |
| Underestimate | 26 | 5.45% | 23 | 7.42%) | 3 | 1.80% |  |  |
| Overestimate | 374 | 78.41% | 245 | 79.03%) | 129 | 77.25% |  |  |

Comparison: Written Measure to Census Data

The following tables show the comparison between the written measure in the COH data to the objective racial composition data from the census. The overall group uses both black and white subjects, and then the results are stratified by race.

| Table S6. Diagnostic statistics for the overall sample and stratified by race for current neighborhood | | | | | | | | | |
| --- | --- | --- | --- | --- | --- | --- | --- | --- | --- |
| **Racial Composition** | **N** | **Sens** | **Spec** | **PPV** | **NPV** | **+LR** | **-LR** | **False + Rate** | **False - Rate** |
| Overall | 421 |  |  |  |  |  |  |  |  |
| Mostly Black |  | 0.6717 | 0.7578 | 0.7112 | 0.7222 | 2.7733 | 0.4332 | 0.2888 | 0.2778 |
| ~ Half Black |  | 0.2143 | 0.8803 | 0.3934 | 0.7556 | 1.7903 | 0.8925 | 0.6066 | 0.2444 |
| Some Black |  | 0.1712 | 0.9452 | 0.5278 | 0.7610 | 3.1241 | 0.8769 | 0.4722 | 0.2390 |
| Black | 304 |  |  |  |  |  |  |  |  |
| Mostly Black |  | 0.6742 | 0.6825 | 0.7500 | 0.5972 | 2.1235 | 0.4774 | 0.2500 | 0.4028 |
| ~ Half Black |  | 0.2169 | 0.8824 | 0.4091 | 0.7500 | 1.8444 | 0.8875 | 0.5909 | 0.2500 |
| Some Black |  | 0.1163 | 0.9579 | 0.3125 | 0.8681 | 2.7625 | 0.9225 | 0.6875 | 0.1319 |
| White | 117 |  |  |  |  |  |  |  |  |
| Mostly Black |  | 0.6500 | 0.8557 | 0.4815 | 0.9222 | 4.5045 | 0.4090 | 0.5185 | 0.0778 |
| ~ Half Black |  | 0.2069 | 0.8750 | 0.3529 | 0.7700 | 1.6552 | 0.9064 | 0.6471 | 0.2300 |
| Some Black |  | 0.2059 | 0.8776 | 0.7000 | 0.4433 | 1.6822 | 0.9049 | 0.3000 | 0.5567 |

| Table S7. Diagnostic statistics for the overall sample and stratified by race for high school | | | | | | | | | |
| --- | --- | --- | --- | --- | --- | --- | --- | --- | --- |
| **Racial Composition** | **N** | **Sens** | **Spec** | **PPV** | **NPV** | **+LR** | **-LR** | **False + Rate** | **False - Rate** |
| Overall | 227 |  |  |  |  |  |  |  |  |
| Mostly Black |  | 0.7544 | 0.6412 | 0.4135 | 0.8862 | 2.1026 | 0.3830 | 0.5865 | 0.1138 |
| ~ Half Black |  | 0.1186 | 0.8095 | 0.1795 | 0.7234 | 0.6226 | 1.0888 | 0.8205 | 0.2766 |
| Some Black |  | 0.1351 | 0.9655 | 0.7895 | 0.5385 | 3.9159 | 0.8958 | 0.2105 | 0.4615 |
| Black | 174 |  |  |  |  |  |  |  |  |
| Mostly Black |  | 0.7544 | 0.5128 | 0.4300 | 0.8108 | 1.5484 | 0.4789 | 0.5700 | 0.1892 |
| ~ Half Black |  | 0.1071 | 0.8305 | 0.2308 | 0.6622 | 0.6319 | 1.0751 | 0.7692 | 0.3378 |
| Some Black |  | 0.0656 | 0.9735 | 0.5714 | 0.6587 | 2.4755 | 0.9598 | 0.4286 | 0.3413 |
| White | 53 |  |  |  |  |  |  |  |  |
| Mostly Black* |  | NA | NA | NA | NA | NA | NA | NA | NA |
| ~ Half Black |  | 0.3333 | 0.7600 | 0.0769 | 0.9500 | 1.3888 | 0.8772 | 0.9231 | 0.0500 |
| Some Black |  | 0.2200 | 0.6667 | 0.9167 | 0.0488 | 0.6601 | 1.1699 | 0.0833 | 0.9512 |
| * No white subjects attended schools that were more than 2/3 black, so diagnostics could not be calculated. | | | | | | | | | |

Tables S8 and S9 show the results for the agreement between the pictorial measure and the census data at the current neighborhood level. The results are stratified by health literacy level.

| Table S8. Pictorial measure versus objective data results for current neighborhood by health literacy level using REALM-R, n=447 | | | | |
| --- | --- | --- | --- | --- |
| **REALM-R Category** | **Agreement** | **N** | **%** | **p-value*** |
| Adequate literacy | Concordance | 79 | 30.74% |  |
|  | Underestimate | 62 | 24.12% |  |
|  | Overestimate | 116 | 45.14% |  |
|  |  |  |  |  |
| Possible likelihood of limited literacy | Concordance | 34 | 30.91% |  |
|  | Underestimate | 40 | 36.36% | 0.1246 |
|  | Overestimate | 36 | 32.73% |  |
|  |  |  |  |  |
| High likelihood of limited literacy | Concordance | 26 | 32.50% |  |
|  | Underestimate | 23 | 28.75% |  |
|  | Overestimate | 31 | 38.75% |  |
| * Corresponds to Chi-square test of independence between concordance and REALM-R category | | | | |

| Table S9. Pictorial measure versus objective data results for current neighborhood by health literacy level using NVS, n=447 | | | | |
| --- | --- | --- | --- | --- |
| **NV Category** | **Agreement** | **N** | **%** | **p-value*** |
| Adequate literacy | Concordance | 46 | 27.38% |  |
|  | Underestimate | 38 | 22.62% |  |
|  | Overestimate | 84 | 50.00% |  |
|  |  |  |  |  |
| Possible likelihood of limited literacy | Concordance | 50 | 34.72% |  |
|  | Underestimate | 45 | 31.25% | 0.0462 |
|  | Overestimate | 49 | 34.03% |  |
|  |  |  |  |  |
| High likelihood of limited literacy | Concordance | 43 | 31.85% |  |
|  | Underestimate | 42 | 31.11% |  |
|  | Overestimate | 50 | 37.04% |  |
| * Corresponds to Chi-square test of independence between concordance and NVS category | | | | |

Tables S10 and S11 show the results for the agreement between the pictorial measure and the census data at the high school level. The results are stratified by health literacy level.

| Table S10. Pictorial measure versus objective data results for high school by health literacy level using REALM-R, n=450 | | | | |
| --- | --- | --- | --- | --- |
| **REALM-R Category** | **Agreement** | **N** | **%** | **p-value*** |
| Adequate literacy | Concordance | 39 | 15.12% |  |
|  | Underestimate | 9 | 3.49% |  |
|  | Overestimate | 210 | 81.40% |  |
|  |  |  |  |  |
| Possible likelihood of limited literacy | Concordance | 22 | 20.00% |  |
|  | Underestimate | 6 | 5.45% | 0.2814 |
|  | Overestimate | 82 | 74.55% |  |
|  |  |  |  |  |
| High likelihood of limited literacy | Concordance | 13 | 15.85% |  |
|  | Underestimate | 7 | 8.54% |  |
|  | Overestimate | 62 | 75.61% |  |
| * Corresponds to Chi-square test of independence between concordance and REALM-R category | | | | |

| Table S11. Pictorial measure versus objective data results for high school by health literacy level using NVS, n=451 | | | | |
| --- | --- | --- | --- | --- |
| **NVS Category** | **Agreement** | **N** | **%** | **p-value*** |
| Adequate literacy | Concordance | 33 | 19.08% |  |
|  | Underestimate | 7 | 4.05% |  |
|  | Overestimate | 133 | 76.88% |  |
|  |  |  |  |  |
| Possible likelihood of limited literacy | Concordance | 23 | 15.86% |  |
|  | Underestimate | 10 | 6.90% | 0.5249 |
|  | Overestimate | 112 | 77.24% |  |
|  |  |  |  |  |
| High likelihood of limited literacy | Concordance | 19 | 14.29% |  |
|  | Underestimate | 5 | 3.76% |  |
|  | Overestimate | 109 | 81.95% |  |
| * Corresponds to Chi-square test of independence between concordance and NVS category | | | | |

The following tables show the agreement results comparing the response on the written measure to the objective data for the subject’s current neighborhood. Tables S12 and S13 use the REALM-R and NVS, respectively, as the measures of health literacy

| Table S12. Written measure versus objective data results for current neighborhood by health literacy level using REALM-R, n=261 | | | | | |
| --- | --- | --- | --- | --- | --- |
| **REALM-R Category** | **Agreement** | **N** | **%** | | **p-value*** |
| Adequate literacy | Concordance | 83 | 61.94% |  | |
|  | Underestimate | 12 | 8.96% |  | |
|  | Overestimate | 39 | 29.10% |  | |
|  |  |  |  |  | |
| Possible likelihood of limited literacy | Concordance | 41 | 57.75% |  | |
|  | Underestimate | 12 | 16.90% | 0.4962 | |
|  | Overestimate | 18 | 25.35% |  | |
|  |  |  |  |  | |
| High likelihood of limited literacy | Concordance | 35 | 62.50% |  | |
|  | Underestimate | 8 | 14.29% |  | |
|  | Overestimate | 13 | 23.21% |  | |
| * Corresponds to Chi-square test of independence between concordance and REALM-R category | | | | | |

.

| Table S13. Written measure versus objective data results for current neighborhood by health literacy level using NVS, n=261 | | | | |
| --- | --- | --- | --- | --- |
| **NVS Category** | **Agreement** | **N** | **%** | **p-value*** |
| Adequate literacy | Concordance | 53 | 61.63% |  |
|  | Underestimate | 10 | 11.63% |  |
|  | Overestimate | 23 | 26.74% |  |
|  |  |  |  |  |
| Possible likelihood of limited literacy | Concordance | 52 | 63.41% |  |
|  | Underestimate | 11 | 13.41% | 0.8897 |
|  | Overestimate | 19 | 23.17% |  |
|  |  |  |  |  |
| High likelihood of limited literacy | Concordance | 54 | 58.06% |  |
|  | Underestimate | 11 | 11.83% |  |
|  | Overestimate | 28 | 30.11% |  |
| * Corresponds to Chi-square test of independence between concordance and NVS category | | | | |

Tables S14 and S15 show the agreement results for the written measure versus objective data for high school racial composition.

| Table S14. Written measure versus objective data results for high school by health literacy level using REALM-R, n=150 | | | | | |
| --- | --- | --- | --- | --- | --- |
| **REALM-R Category** | **Agreement** | **N** | **%** | **p-value*** | |
| Adequate literacy | Concordance | 31 | 39.24% | |  |
|  | Underestimate | 1 | 1.27% | |  |
|  | Overestimate | 47 | 59.49% | |  |
|  |  |  |  | |  |
| Possible likelihood of limited literacy | Concordance | 15 | 38.46% | |  |
|  | Underestimate | 1 | 2.56% | | <0.0001 |
|  | Overestimate | 23 | 58.97% | |  |
|  |  |  |  | |  |
| High likelihood of limited literacy | Concordance | 11 | 34.38% | |  |
|  | Underestimate | 6 | 18.75% | |  |
|  | Overestimate | 15 | 46.88% | |  |
| * Corresponds to Chi-square test of independence between concordance and REALM-R category | | | | | |

| Table S15. Written measure versus objective data results for high school by health literacy level using NVS. N=151 | | | | |
| --- | --- | --- | --- | --- |
| **NVS Category** | **Agreement** | **N** | **%** | **p-value*** |
| Adequate literacy | Concordance | 21 | 42.86% |  |
|  | Underestimate | 0 | 0% |  |
|  | Overestimate | 28 | 57.14% |  |
|  |  |  |  |  |
| Possible likelihood of limited literacy | Concordance | 19 | 38.00% |  |
|  | Underestimate | 1 | 2.00% | <0.0001 |
|  | Overestimate | 30 | 60.00% |  |
|  |  |  |  |  |
| High likelihood of limited literacy | Concordance | 18 | 34.62% |  |
|  | Underestimate | 7 | 13.46% |  |
|  | Overestimate | 27 | 51.92% |  |
| * Corresponds to Chi-square test of independence between concordance and NVS category | | | | |

Comparison of the written and pictorial measures: Tables S16 and S17 show the results comparing the agreement between the written and pictorial racial composition measures for each level of health literacy.

| Table S16. Written and pictorial agreement and health literacy using REALM-R | | | | |
| --- | --- | --- | --- | --- |
|  | **% with Written-Pictorial Concordance** | | |  |
| **Location** | **Adequate Literacy** | **Possible Likelihood of Limited Literacy** | **High Likelihood of Limited Literacy** | **p-value** |
| Current neighborhood | 68.39% | 56.96% | 60.94% | 0.1982 |
| High School | 71.43% | 69.23% | 70.69% | 0.9423 |

| Table S17. Written and pictorial agreement and health literacy using NVS | | | | |
| --- | --- | --- | --- | --- |
|  | **% with Written-Pictorial Concordance** | | |  |
| **Location** | **Adequate Literacy** | **Possible Likelihood of Limited Literacy** | **High Likelihood of Limited Literacy** | **p-value** |
| Current neighborhood | 70.00% | 64.58% | 56.86% | 0.1486 |
| High School | 71.59% | 71.84% | 68.09% | 0.8173 |
